# A Report of Some Rare Surgical Lesions Connected with the Liver*Read before Tri State Medical Society of Alabama, Georgia, and Tennessee, October, 1894.

**Published:** 1894-10

**Authors:** John A. Wyeth

**Affiliations:** New York


### Southern Medical Record.

A MONTHLY JOURNAL OF MEDICINE AND SURGERY.

VOL. XXIV. ATLANTA, GA., OCTOBER, 1894. No. 10.

### Original Articles.

### A REPORT OF SOME RARE SURGICAL LESIONS CONNECTED WITH THE LIVER.*

BY JOHN A. WYETH, M.D., NEW YORK.

#### OBSTRUCTION OF THE HEPATIC, CYSTIC, AND COMMON DUCTS.

CASE I.—Mrs. E., fifty years, housewife, under observation April 10, 1894. Family history good. In June, 1893, had well marked attack of hepatic colic. For six months before this date she had been ailing, but presented no special symptoms of liver disease.

With her first attack she became jaundiced, and this condition prevailed without interruption to the day of her death. She has had repeated attacks of colic, each followed by excessive tenderness over the region of the gall-bladder and duodenum. No gall stones have ever been found in the discharges, and these have been carefully watched for.

On examination, patient profoundly jaundiced; complained of dyspnea and incessant headache. No tenderness anywhere, and no distended gall-bladder recognized. Liver considerably enlarged, extending about one and one-half inches below the costal

*Read before Tri State Medical Society of Alabama, Georgia, and Tennessee, October, 1894.

arch. Heart and lungs normal. Urine deeply stained with bile, otherwise normal.

Operation April 12th, in the Polyclinic Amphitheater—ether—long incision parallel with, and one inch below right costal arch. Extensive adhesions between colon, omentum, and small intestines and to gall-bladder. This was very deeply situated, small, elongated, and with thick walls. It was about five inches long, about one inch in diameter at the summit, and gradually tapered in a cone to the apex at the cystic duct. The bladder, near the exit of the cystic duct, this duct, and the hepatic and common ducts were obstructed by a mush-like substance which gave way easily to the pressure of the finger.

I incised the bladder, protecting the general peritoneal cavity by sterilized napkins, and through this opening extracted, by squeezing, a putty-like substance, in all about a teaspoonful.

The common duct was emptied by stripping it toward the intestine, pressing its soft contents into the duodenum. Near the junction of the cystic and hepatic ducts was a harder nodule, which could not be displaced. I incised the duct over this, and removed it. A lot of detritus was now removed by irrigation of the duct and bladder through one opening, and out at the other. Hot boric acid solution was used. Fine sutures of iron dyed silk were inserted and both the incision in the cyst and duct closed.

While breaking up adhesions in the earlier part of the operation, a long branch of the hepatic artery was torn in two and was with some difficulty caught with a long ovarian sac straight forceps, and tied.

The entire time of operation was two hours. The patient came out of the narcosis promptly and gave no evidence of shock. Three hours after the operation six ounces of urine were drawn by catheter. Four hours later symptoms of uremia were present. Suppression of urine was soon complete, coma supervened, and despite the early and energetic employment of digitalin, and stimulants, the patient perished thirty-six hours after operation.

The autopsy revealed complete occlusion of both branches of the *hepatic* duct up to their exit from the right and left lobes of the liver. The occluding material was the same soft substance squeezed out of the common duct. The sutures were tested and found to be water-tight.

OBLITERATION OF HEPATIC DUCT.

CASE II.—P. R., female, thirty-five years old, Russian, mother of nine children, the youngest six weeks old when I saw her, December 1, 1893. About the third month of her last pregnancy, she was seized with pains or cramps in the epigastric region, and at the same time she became jaundiced. The jaundice has remained, and she has frequent attacks of pain. There is no history of chill or fever. Bowels reported as regular, but there is complete acholia. No peritonitis. Last week she had slight cough. General condition fairly good, except the jaundice. Tongue coated, lungs and heart normal, breasts secreting. Some tenderness over the right hypochondriac region. No tumor.

December 4th, operation—ether—incision parallel with free border of the ribs over the location of the gall-bladder. Upon entering the abdomen, no gall-bladder could be felt. Extensive adhesions between the small intestine, gastro-hepatic omentum, and the colon.

After tearing away the numerous adhesions, the gall-bladder was found to be collapsed and small, measuring not more than an inch in its largest diameter, completely empty, and flattened down like a Tam O'Shanter cap. No calculus in either of the ducts. The patient recovered from the operation, but died on the 10th of December, six days later, from persistent cholæmia. At the autopsy a biliary calculus, one-fourth of an inch in diameter was found floating in the peritoneal cavity near the cecum. The gall-bladder was found as described, and there was a complete obliteration of the hepatic and cystic ducts.

Cicatricial contraction at the junction of the hepatic with the cystic duct pointed clearly to the former location of the calculus, which had cut its way through at this point into the free peritoneal cavity, and the resulting cicatrization had closed the hepatic and cystic duct.

TRAUMATIC RUPTURE OF THE GALL-BLADDER—GENERAL ESCAPE OF BILE INTO THE PERITONEAL CAVITY—OPERATION TWENTY-FOUR HOURS LATER.

CASE III.—An Italian laborer about twenty-five years of age, walked into the clinic at the New York Polyclinic, with the following history:

His general condition was good, having been working daily as a laborer on some of the public works of the city. On the day previous, he had been struck over the right hypochondriac region by a piece of timber. He suffered some shock at the time, but after taking some stimulants, reacted, and went to his home, and the next day he was advised to apply at the Polyclinic for advice and treatment. At that time, the abdomen was considerably distended, and evidently contained fluid. Upon careful aspiration, with a very small hypodermic needle, a dark colored fluid with a yellowish tinge was drawn off. A diagnosis of rupture of the gall-bladder was made from the characteristic stain of the fluid, and immediate operation advised. The patient was put under the influence of an anesthetic. A longitudinal incision was made over the location of the gall-bladder, and upon opening the peritoneal cavity, the gall-bladder was clearly in view. It was found to be lacerated for about an inch in extent, and contained a small amount of fluid. The intestines and the general contents of the peritoneal cavity were stained with extravasated blood and bile.

The torn edges of the gall-bladder were brought up to the edges of the wound in the abdomen, and stitched with silk sutures, and drainage inserted into the gall-bladder. The balance of this wound was closed. An incision was then made in the median line of the abdomen, half-way between the umbilicus and the pubes, and the peritoneal cavity thoroughly washed out by a continuous irrigation of water which had been boiled and allowed to cool to 110° F.

A draining tube was inserted leading through this wound from the deepest portion of the pelvic cavity. This was removed thirty-six hours later. The patient required no further treatment, and made prompt recovery. The fistulous opening in the gall-bladder closed spontaneously at the end of about two weeks.

CASE V.—J. P., male, thirty-six years of age. Came under my observation in August, 1893. He had been a healthy man up to October, 1892. At that time he began to notice a slight enlargement in the region of the liver, and at the same time to observe that his general health was impaired, and that fluid was accumulating in lower part of the peritoneal cavity. The fluid gradually increased to such an extent that he had to be tapped. This operation was repeated at intervals, from ten days to two or three weeks, for eleven months prior to the date at which I first saw him, August, 1893. At each one of these tappings, from three to four gallons of serous fluid were removed. When he came under my care, his condition was exceedingly bad. He was sallow, emaciated, so weak that it was with difficulty that he could move himself across the room without help. I removed four gallons of fluid from him on two occasions, only twelve days between the two operations.

When the abdominal cavity was empty, a large tumor, seemingly attached to the left lobe of the liver and the gastro-hepatic omentum, extending downward to the median line, measuring from six to nine inches in its largest diameter, could be distinctly made out.

Taking into consideration the patient's cachexia, which pointed to occlusion of the portal vein, it seemed to me that this was a case of carcinoma of the liver or of the duodenum, or some of the organs situated near the point of entrance of the portal vein into the liver. For some months prior to this time, my attention had been directed toward the effect of the toxine of erysipelas, as well as of the ordinary suppurative processes, upon these malignant neoplasms.

I suggested to this gentleman the advisability of submitting to inoculation of one of these toxic products in view of his hopeless condition, to which, after due consideration, he assented.

In August, 1893, I opened the abdominal cavity immediately over the tumor, and found that I could attach the surface of the neoplasm to the margins of this incision, allowing it to become infected with the ordinary coccus of suppuration.

The wound discharged a considerable quantity of pus, was kept opened and drained for two months. The patient improved from the first week after the operation, and has continued to improve to this date. He has never been tapped since the operation, and although the neoplasm can still be distinctly outlined, it has never enlarged. The patient is able to attend to his active business affairs, and does not seem to be inconvenienced by its presence. He has gained about twenty pounds in weight since the operation was performed.

CASE IV.—A Mexican lad sixteen years of age, attending school in New York, was, while coasting in February, 1894, thrown violently against a tree, striking on the left side over the region of the tenth, eleventh, and twelfth ribs. He suffered considerable shock for two or three hours, but finally reacted well, and, while complaining of considerable soreness for ten or twelve days, gave no symptom of deep internal injury beyond the passage of bloody urine for two or three days immediately after the accident.

From this time the patient seemingly recovered and was up and about at the end of five weeks. About this time, while riding on the rear of an ice wagon, he fell to the ground, experienced a sharp pain in the region where the former injury had been received, went home and went to bed. Some weeks after this, his attending physician called a surgeon in consultation.

It was deemed proper to explore the region of the left kidney, but no injury to this organ was discovered, and although high temperatures had existed for several days, pointing to the presence of pus, no suppuration was found.

On the 29th day of May I was called in consultation with the attending physicians, and after a careful examination of the lad under ether, we established the presence of pus beneath the diaphragm, and between this organ and the liver. At the request of Dr. Goffe and Dr. Wanzer, the physicians in charge of the patient, the following operation was performed:

An incision was made immediately over the eighth rib on the right side about half-way between the vertebral column and the sternum. Three inches of this rib were removed without entering the thoracic cavity. A clean aspirator needle was then passed through the costal pleura traversing the thoracic cavity to the diaphragm, then through the diaphragm, when it entered the cavity of the abscess. Enough of the contents of the cavity were aspirated to prevent the overflow of pus into the thoracic cavity, while the operation was proceeding.

The aspirator needle was left in position, and the costal pleura opened for the full extent of the wound—three inches. The edges of the costal pleura were stitched by continuous silk suture, to the diaphragm, care being taken to insert the suture in the expiratory act, and to close the wound during inspiration in order to prevent the entrance of air into the thoracic cavity.

The operation being completed, the diaphragm was incised; a large quantity of pus evacuated through irrigation, and ordinary dressing applied.

The patient rallied from the operation and improved considerably for several days. About a week later, the wound ceased to discharge any more pus, and the patient immediately gave evidence of septic absorption by high temperature.

I was called again to see him in this condition, and thought it inadvisable to operate further, owing to his extreme weakness, and retired from the case. I am informed that the boy died within twelve hours of this last visit. At the autopsy, a large sub-diaphragmatic abscess was found, which involved a large portion of the spleen and the space between the upper surface of the liver and the diaphragm, the two abscesses connected by a narrow sinus along the under surface of the diaphragm. The original lesion had doubtless been a laceration of the spleen with an insignificant laceration of the kidney. The passage of bloody urine being the only symptom that could be positively recognized, directed the attention of the first surgeon called to the left kidney as the possible seat of lesion. The abscess had doubtless formed first in the spleen and between this organ and the diaphragm, and had burrowed along the under surface of the diaphragm to the liver.

Of these five cases, the first two, being cases of obstruction of the hepatic duct, show how utterly hopeless surgical interference must be in the effort to overcome the obstruction. While, as is well known, obstructions of the common duct can be overcome in the vast majority of cases, the deep situation of the hepatic duct and its small size render incision into it and a subsequent suture of this duct practically impossible.

This case I have reported on account of its unusual nature, and it is unique in my experience. The case of occlusion as a result of the escape of gall stone at the junction of the hepatic and cystic ducts, is also unusual, and while many cases are on record in which biliary calculi have escaped from the gall-bladder into the intestines, such escapes into the general cavity of the peritoneum are rare.

It is an observation of interest that no septic peritonitis was established by the escape of this foreign body and its wandering through the peritoneal cavity, and no doubt had the occlusion of the hepatic duct been complete, the patient would have recovered and the wandering calculus would have been lost sight of.

As to the third case, rupture of the gall-bladder and the escape of its contents into the peritoneal cavity, it is not so very uncommon. Several cases are on record to my knowledge. The prognosis in case of such an accident must, of course, depend upon the aseptic nature of the fluid contents of this organ. If it is pure bile, which in itself is an aseptic fluid, the danger of septic peritonitis is insignificant. If, however, there is a suppurative cystitis of the gall-bladder, containing a septic pus, the infection must of necessity become general.

The fourth case reported is one of the most surprising experiences in my professional life. That a man with a large, malignant tumor of the liver, already developed to such an extent that compression of the portal vein was evident with general ascites, could, as a result of an infection from the streptococcus pyogenes aureus, undergo such a change within a few weeks of time that this pressure on the portal vein was removed, is a matter of astonishment to myself. It brings up, naturally, consideration of the action of various toxic products upon the new tissues which form certain tumors, such as carcinoma and sarcoma. It is a fact now established in surgical pathology that the action of Fehleisen's coccus and other toxic products or ptomaines are capable of causing a disappearance of that group of neoplasms classed as sarcoma. I myself have reported several cases of sarcoma in which a diagnosis has been made, without doubt, and in which, in one instance, an acute suppurative non-erysipelatous process caused the entire disappearance of a large sarcoma of the abdominal wall, a period of ten years having elapsed without recurrence, and the patient is now living in perfect health, weighing, as I have been informed recently, one hundred and seventy pounds. Similar cases are so well established, and are so well known in surgical literature that a further consideration is unnecessary.

The action of the poison of erysipelas itself upon the presence of epithelioma is also well established. I have reported one case of epithelioma which disappeared after an attack of erysipelas which involved the area on which the epithelioma was located. As to the action of these toxines upon adenoid carcinoma, our information is, as yet, not positive. I firmly believe that since one form of malignant neoplasm, which must have its specific cause, is cured by one form of toxine, ultimately all other forms of tumors will find their specific remedy and be cured in like manner by inoculation.

There is nothing of special interest in the fifth case, to my mind, beyond the surgical technique, and I have reported it simply to emphasize the necessity for uniting the anterior costal pleura to the pleural covering of the diaphragm, in this way absolutely shutting off the pleural cavity from general infection in opening sub-diaphragmatic abscesses, whether they be over the liver or over the spleen. Certainly no greater disaster could follow an abscess in this region, which cannot be opened below the diaphragm for fear of general peritoneal infection, than to have a septic process established in the pleural cavity by an unfortunate operative result.

---

### THE SLAUGHTER OF THE INNOCENTS.*

BY DR. E. VAN GOIDTSNOVEN, ATLANTA, GA.

We are constantly reminded that all living things on this our planet are subject to the laws of death; that this earth is an immense crematory; that we live upon the ashes of the dead; that life within us presides over the perpetual conflict between anabolic and katabolic agencies; and that metabolism is the *ultima ratio* of all disintegration, of all oxidation; that molecular death is incessantly wrought throughout our economy; that death of the individual is

